# Melatonin’s neuroprotective role in mitochondria and its potential as a biomarker in aging, cognition and psychiatric disorders

**DOI:** 10.1038/s41398-021-01464-x

**Published:** 2021-06-02

**Authors:** Lindsay M. Melhuish Beaupre, Gregory M. Brown, Vanessa F. Gonçalves, James L. Kennedy

**Affiliations:** 1 Molecular Brain Science Research Department, Campbell Family Mental Health Research Institute, Centre for Addiction and Mental Health, Toronto, ON Canada; 2 Institute of Medical Sciences, University of Toronto, Toronto, ON Canada; 3 Department of Psychiatry, University of Toronto, Toronto, ON Canada

**Keywords:** Molecular neuroscience, Clinical genetics

## Abstract

Melatonin is an ancient molecule that is evident in high concentrations in various tissues throughout the body. It can be separated into two pools; one of which is synthesized by the pineal and can be found in blood, and the second by various tissues and is present in these tissues. Pineal melatonin levels display a circadian rhythm while tissue melatonin does not. For decades now, melatonin has been implicated in promoting and maintaining sleep. More recently, evidence indicates that it also plays an important role in neuroprotection. The beginning of our review will summarize this literature. As an amphiphilic, pleiotropic indoleamine, melatonin has both direct actions and receptor-mediated effects. For example, melatonin has established effects as an antioxidant and free radical scavenger both in vitro and in animal models. This is also evident in melatonin’s prominent role in mitochondria, which is reviewed in the next section. Melatonin is synthesized in, taken up by, and concentrated in mitochondria, the powerhouse of the cell. Mitochondria are also the major source of reactive oxygen species as a byproduct of mitochondrial oxidative metabolism. The final section of our review summarizes melatonin’s potential role in aging and psychiatric disorders. Pineal and tissue melatonin levels both decline with age. Pineal melatonin declines in individuals suffering from psychiatric disorders. Melatonin’s ability to act as a neuroprotectant opens new avenues of exploration for the molecule as it may be a potential treatment for cases with neurodegenerative disease.

## Introduction

Melatonin is a pleiotropic indoleamine that is amphiphilic so that it can readily cross from blood or cerebral spinal fluid (CSF) into tissues and cells, as well as through the blood–brain barrier.

For years now, circulating melatonin has been well-known to promote sleep, maintain sleep, reset the circadian clock, and entrain free-running circadian rhythms^1–7^. However, melatonin and its derivatives are now recognized to also have very potent effects as free radical scavengers and antioxidants^8^. Melatonin is present very widely in body tissues and in almost all of them the synthesizing enzymes arylalkylamine N-acetyltransferase (AANAT) and acetylserotonin O-methyltransferase (ASMT) have been found^9^. Because mitochondria are the powerhouse of the body, synthesizing ATP via oxidative phosphorylation, the presence of melatonin was sought and found in the organelle^10^. In fact, mitochondria from rodent maternal oocytes can synthesize melatonin from serotonin, which is consistent with the fact that mitochondria are maternally derived^11,12^. Thus, this powerful antioxidant has a significant protective presence in the body’s major source of free radicals^13–15^.

We aim to provide a brief overview of melatonin and its neuroprotective role, with an emphasis on mitochondrial melatonin. Due to the plethora of evidence implicating mitochondria in the aging process, as well as psychiatric disorders^16^, we will provide a succinct discussion of melatonin’s potential role as a factor and marker of aging and psychiatric disorders to close off this review.

## History and overview of melatonin

Melatonin is an ancient molecule, found in bacteria, plants, and molds^17^. In various species, before any hint of a hormonal role it had a local regulatory function^18^. In reptiles and birds, it was present in several locations including the eyes. The third parietal eye which served as a sensor for the presence of light was one of those eyes containing melatonin^19^.

There is evidence that the primitive third eye evolved into the pineal gland in mammals^20^. It remained linked to the light-sensing system by a neural link but then passes that information on by the neuroendocrine signal, melatonin. In body tissues, it is found in high concentrations in numerous tissues including the harderian gland, retina, hypothalamus, liver, colon, the entire gastrointestinal tract, and immune system^9,19,21–25^. These two systems, hormonal and tissue are separate pools. It has been known since 1980 that gastrointestinal tissue levels are independent of blood levels; pinealectomy does not lower tissue levels, but abolishes virtually all blood levels^23,26^. One pool is synthesized in the pineal, the other is present in virtually every body tissue^9,27–29^. The pool of tissue melatonin is far greater (10–400×) than that derived from the pineal gland^26,30,31^.

Pineal melatonin levels in plasma and serum display a circadian (about 24 h) rhythm in which levels are vanishingly low during the day and increase during the dark period, peaking around 2–4 a.m. before dropping again^32–34^. Synthesis and secretion of melatonin are controlled by the suprachiasmatic nucleus (SCN), the master clock of the body. The SCN contains a set of genes that interact in a self-contained transcription–translation negative feedback loop with a loosely 24-h cycle^35,36^. Lesioning the SCN eliminates endogenous melatonin rhythmicity and produces an inability for exogenous melatonin to resynchronize the system^37,38^. This rhythm is synchronized with the light–dark (LD) cycle through input from the retina via the retinohypothalamic tract, which arises from a tiny set of innately photosensitive ganglion cells (IPGCs). These IPGCs contain the photopigment melanopsin, which is particularly sensitive to light in the blue spectrum. These neurons convey information on the LD cycle to the SCN, to regions that regulate pupillary responses as well as to sleep and waking systems^39^. Projection to the pineal is multi-synaptic initially to the autonomic section of the hypothalamic paraventricular nucleus, then leading to a projection to the upper thoracic intermediolateral cell column. From there, preganglionic sympathetic noradrenergic fibers travel to the superior cervical ganglion that sends postganglionic fibers to the pineal gland, thus initiating melatonin synthesis. There is an extremely rapid response in AANAT to produce N-acetylserotonin, increasing 10–100-fold during night-time^40^. That substance is then converted to melatonin by the enzyme ASMT [formerly called hydroxyindole O-methyl transferase (HIOMT)]^41^. Melatonin is not stored, being secreted directly into the blood stream where it is largely bound to albumen. Melatonin measurement in CSF shows that content in the third ventricle is not only higher than in the lateral ventricle but also higher than in plasma, indicating that there is direct entry from the pineal to CSF and not just from the blood in the choroid plexus is probable^42,43^.

Two G_1_-protein linked melatonin receptors MT1, and MT2 are known^44,45^. Like other G_1_-protein-linked receptors (GPCR), they frequently become associated as dimers; the heterodimer MT1/MT2 is as frequent as the homodimer of MT1, while the homodimer of MT2 is almost 4-fold less common. A third receptor, GPR-50, has a sequence that is 45% related but will not bind melatonin. However, it will form heterodimers with MT1 that abolish binding and may therefore be functionally significant. Yet a fourth related mammalian melatonin binding site has been found. It has nanomolar rather than picomolar affinity for melatonin and has now been characterized as the analog of quinone reductase type 2 in hamster kidney^46^. Both MT1 and MT2 receptors are present in the SCN. MT1 inhibits firing, while both may cause phase shifting and differentially regulate GABA_A_ function^47,48^. Both MT1 and MT2 receptors are widely distributed in the brain and appear to have differential functions in rapid eye movement (REM) versus non-REM sleep, anxiety, and vigilance^49–53^. Both receptors are also found in many other parts of the body and have been shown to mediate/activate some of melatonin’s neuroprotective effects^54,55^.

## Melatonin and neuroprotection

There is ample evidence to support melatonin’s role in neuroprotection. The concept was first established by Tan et al. (1993)^56^ who discovered its ability to scavenge for free radicals, more specifically, hydroxyl radicals in vitro^56^. The concept of melatonin being able to scavenge for free radicals was further shown both in vitro and using animal models^57,58^. In fact, animal studies have found that melatonin is effective in scavenging free radicals during both postischemic reperfusion and after head trauma^59,60^. It should be noted that the time of melatonin administration is critical when treating head trauma. Melatonin only reduces malondialdehyde, a marker of oxidative stress, when melatonin was administered within the first two hours post-trauma. If given 8 h or even 48 h after the injury has occurred, then melatonin only increases malondialdehyde levels, though the reason why remains unclear^60,61^. Interestingly, Zang et al. (1998)^62^ were unable to replicate the results with hydroxyl radicals. They postulate that this negative finding was because all experiments performed were in the presence of hydrogen peroxide, for which melatonin is a dose-dependent scavenger^62^. However, increasing melatonin levels causes greater scavenger capabilities^62^.

Serum melatonin also has been shown to exhibit an antioxidant capacity, and accordingly, the peak in antioxidant capacity is dependent on the surge in melatonin^63^. Antioxidant capacity may also have important implications for neurocognition in those with depressive disorder^64–66^.

Melatonin can also be found throughout the immune system and is now known to also be an immune modulator, one with a double action^67^. On the one hand, it boosts immunity against foreign invasion while on the other hand it modulates tissue responses, downregulating proinflammatory and up-regulating anti-inflammatory cytokines. Melatonin has been shown to improve morbidity and mortality both in sepsis in animals and in children^68^. It has also been shown to have a very large safety margin and in animals, the administration has never been fatal when given orally or subcutaneously, hence the LD50 has been stated to be infinity^68,69^.

In a recent comprehensive review, it was pointed out melatonin acts both through receptor-dependent and independent pathways to protect against neurodegeneration^55^. For example, agomelatine, a non-specific MT1/2 receptor agonist is used to treat major depressive disorder (MDD), and it also improves sleep patterns and normalizes circadian rhythms^54,70,71^. In addition, administration of melatonin to MT1/2 knock-out mice following a brain perfusion (to induce focal cerebral ischemia) led to some neuroprotection, as measured by the reduction of infarct volume^72^. Melatonin receptors may also play a vital role in protecting against neurodegeneration. In the human SH-SY5Y cell line (that has protein expression similar to that of Alzheimer’s disease), it was shown that melatonin administration inhibited β-secretase β-site APP-cleaving enzyme 1 (BACE1) and Prensenilin 1 (PS1) expression while increasing a disintegrin and metalloproteinase 10 (ADAM10), each of which is involved in the formation of Alzheimer-related amyloid β-peptides. All alterations found in BACE1, PS1, and ADAM10 were receptor-mediated; administration of a G protein inhibitor before the melatonin treatment abolished the effects of melatonin. This highlights the importance of the melatonin receptors in inhibiting neurodegeneration via the activation of melatonin^73^. However, there is an abundance of receptor-mediated neuroprotective effects that are amply reviewed recently elsewhere (please see refs. ^74,75^) so the remainder of the review will be focused on mitochondrial-mediated actions.

## Melatonin and mitochondria

Importantly, melatonin displays neuroprotective effects on mitochondria via its free radical scavenging capabilities. For example, it has been shown that the administration of melatonin protects against mitochondrial DNA (mtDNA) damage that is potentially induced by ROS^76^. Administration of melatonin to a pregnant mother rat also increases the activity of glutathione (GSH) peroxidase, an antioxidant marker, in fetal brains^77^. Mitochondria found in the brain and liver contain high amounts of melatonin^23,78^. Martin et al. (2000)^79^ found that a 100 nanomolar dose of melatonin given to mitochondrial membranes from rat brain and liver produces intramitochondrial levels that are 100 times greater than the levels in plasma. Given mitochondria’s role in the production of ROS, it makes sense that the highest concentration of melatonin would be in the mitochondria, the site of mitochondrial oxidative metabolism. This means that the greatest amount of ROS and oxidative stress occurs at a site where melatonin is highest, and thus is in an ideal position to act as a scavenger of these free radicals^23^.

It has been hypothesized that the high levels of melatonin in mitochondria can be attributed to (1) oligopeptide transporters (PEPT1/2) and/or (2) mitochondria synthesize their own melatonin^78^. In fact, a recent study found that two enzymes involved in melatonin synthesis, AANAT and ASMT were present in brain mitochondria^10,12,13,80^. However, it is also important to note that the melatonin levels in mitochondria do seem to reach a saturation point^23^. If melatonin can reach saturation, does that mean its free radical scavenging activity can also reach a maximum? To the best of our knowledge, this has yet to be investigated.

In addition to its antioxidant activity, melatonin promotes activities of antioxidant enzymes and reduces pro-oxidant enzymes^78^. One example of an antioxidant enzyme is GSH whose synthesis is stimulated by melatonin^81^. The activity of the antioxidant enzyme, superoxide dismutase 2 (SOD2) is upregulated by melatonin through the promotion of the activity of sirtuin 3 (*SIRT3*) which deacetylates SOD2, thus activating it^82,83^. It should be noted that the half-life of highly reactive ROS is very, very short (e.g. for –OH, 10^−9^ s) so that they travel extremely short distances before oxidizing adjacent molecules^81^. Thus, the juxtaposition of antioxidants and scavengers with the site of ROS production in mitochondria, as is the case for melatonin and its secondary effects, is essential for them to be highly effective.

Melatonin’s effects on mitochondria can be directly mediated via the MT1/2 receptors. For example, treating rats with agomelatine after a cerebral ischemia, led to reduced ROS production in the brain, greater antioxidant properties, and less neuronal apoptosis because of an increase in nuclear factor erythroid 2-related factor 2 (*NRF2*)^84^. Melatonin activates NRF2, which is considered a defense mechanism against ROS as it controls the expression of a collection of genes involved in antioxidant defenses and inflammatory responses^85–88^. Melatonin treatment prevents apoptosis and mitochondrial damage caused by hydrogen-peroxide in retinal pigmented epithelial cells via the activation of melatonin through the MT1 receptor^89^. Remarkably, it has also been shown that the melatonin receptor MT1 is present on mitochondrial outer membranes and that melatonin acts on that receptor to inhibit stress-mediated cytochrome C release, thereby highlighting another neuroprotective property of melatonin^10^.

## Melatonin levels as a potential biomarker?

Unfortunately, melatonin levels do not remain constant throughout life or may become altered. This is seen during aging and in individuals with psychiatric disorders^90–92^. These will be discussed below.

### Aging and age-related cognitive decline

A substantial literature has demonstrated that melatonin levels are known to decline with age^92–96^. The putative effects attributed to these changes may therefore be related to changes in either pool of melatonin^97,98^. Urinary analyses found, on average, individuals between 20 and 39 years old excrete about 12 micrograms of 6-sulphatoxymelatonin (6SMT), the primary metabolite of melatonin, and that this steadily declined to about 6 μg in some individuals over 80^94^. In fact, it has been found that daytime melatonin levels in CSF drop by about half between the ages of 15 and 50^92^. Looking across the entire life span, nocturnal serum melatonin levels appear low during the first 6 months of life, then they peak at 1–3 years of age. By 15–20 years old individuals already experience, on average, an 80% decline in melatonin levels and this decline continues into old age (70–90 years)^95^. Younger individuals also experience their peak melatonin secretion later in sleep than older individuals^99,100^. Perhaps this is because melatonin secretion is correlated with the participants’ habitual bedtimes, which is later for younger adults^99^. Another study found that nocturnal serum melatonin levels are significantly different between individuals <60 and those over 60 years of age, when multiple samples are drawn throughout the night. When only one sample was looked at (2:00 a.m.), the differences were abolished^96^. Daytime serum levels also display mixed results. One study found that daytime serum levels display a negative correlation with age but another study was unable to replicate this finding^93,96^.

There are also instances where the correlation between melatonin levels and aging was not seen at all. Zeitzer et al. (1999)^101^ postulate that their negative findings in plasma were because both their younger and older participants underwent extensive medical examining and were free of diagnoses, medications, nicotine, alcohol, and caffeine, steps that were not documented by other studies. The study by Zeitzer et al. (1999)^101^ also only included individuals between the ages of 18 and 81, whereas most of the other studies included individuals outside of that age range^93,94,96^. One thing to note about all of this research is that melatonin levels vary person-to-person and all of these studies utilize a cross-sectional design^102–105^. This person-to-person variation may be partially explained by genetics^106^.

Animal studies have also found that age-related changes are not only in melatonin derived from the pineal but also in tissue melatonin. Decreased mRNA activity of AANAT and ASMT were found in situ^107^. Decreased AANAT mRNA levels were evident in the spleen and liver of 12-month-old rats (compared to 3-month-old rats) while decreased ASMT levels were present in the spleen only. Increased mRNA expression levels of both enzymes were found in the heart. Moreover, increased AANAT enzyme activity was found in the liver and kidney which the authors suggest may be a compensatory mechanism^107^.

According to the Free Radical Theory of Aging proposed by Harman, free radical reactions produce free radicals, such as ROS, which contribute to the aging process via oxidative changes including damage to nuclear DNA and mtDNA^108^. mtDNA is three times more susceptible to oxidative stress which can lead to mitochondrial dysfunction and apoptosis^109^. This is because mtDNA lacks histones and due to its proximity to the electron transport chain^110^. Antioxidants such as melatonin and others found in mitochondria (e.g. GSH peroxidase) are defenses that have been developed over time to either directly scavenge the free radicals or indirectly metabolize them or their intermediates to neutralize them, thus preventing the deleterious effects they may cause^110–113^. Although other factors, such as mitochondrial transcription factor A may also be important^15^. Unfortunately, aging also leads to a decline in total antioxidant capacity in parallel with melatonin decline^63^.

Another frequent consequence of aging is cognitive decline. This decline in cognition has been linked to both an increase in oxidative stress and a decrease in pineal melatonin levels. For example, a recent study found decreased levels of GSH at baseline, which is indicative of greater oxidative stress and a decline in executive functioning over 4 years^114^. In another study, individuals with dementia experienced a flattening in the circadian curve of plasma melatonin levels compared to mentally healthy individuals of the same age^115^. Furthermore, the nocturnal plasma melatonin peak was significantly associated with cognitive impairment, as determined by the Mini Mental State Examination^116^. There are also reported differences in salivary melatonin levels. Waller et al. (2016)^117^ separated individuals based on their Draft board intelligence scores; individuals who scored remarkedly high were classified as the cognitively high-functioning group, and those who scored low were classified as the cognitively impaired group. Using saliva samples that were collected over a 24-h period, they noticed that the median nocturnal melatonin response at 4 a.m. was significantly lower in the cognitively impaired group. However, there were no significant differences at any other time point^117^. The question then becomes: would exogenous melatonin be of benefit? Although the question cannot be answered directly, we do have some insight from animal models. For example, mice exposed to formaldehyde suffer from cognitive impairments and experience an increase in oxidative stress, as noted by higher levels of ROS, 50% reduction in GSH, and decreased endogenous melatonin. However, melatonin treatment was able to ameliorate the reduction in GSH, restore melatonin levels and improve cognitive functioning^118^. Taken together, this evidence supports a decline in melatonin and an increase in oxidative stress during cognitive decline, independent of age. It also suggests that exogenous melatonin may be beneficial in combatting these changes but further research into this matter is warranted. In a more recent study, melatonin and nicotinamide mononucleotide (NMN) separately or together reversed age-related cognitive impairments and reduced the mitochondrial ROS produced in the prefrontal cortex and hippocampus of aging rats^119^. NMN is the precursor to nicotinamide adenine dinucleotide, which plays a pivotal role in OXPHOS. Overall, the literature suggests that the relationship between reduced melatonin and increased oxidative stress is a complex one that requires further study.

#### Psychiatric disorders

This section reviews the literature on pineal melatonin. To the best of our knowledge, there have been no studies on tissue melatonin levels in any psychiatric disorder as of yet.

#### Major depressive disorder

For decades, decreased nocturnal melatonin levels have been reported in both serum and plasma, implying lower nocturnal secretion in MDD individuals^120–123^. There is, however, inconsistency in morning levels as one study found they, too, were decreased while a second study found they were actually increased in MDD individuals^121,124^. In healthy individuals, reduced nocturnal melatonin levels have been linked to poorer sleep quality, including REM sleep alterations^125^. Interestingly, these altered sleep patterns are also present in MDD patients^126^. No alterations in melatonin levels in the CSF have been identified in MDD patients2^124^.

#### Schizophrenia

Several studies, including a recent meta-analysis have reported that individuals with schizophrenia have decreased nocturnal melatonin in both serum and plasma, regardless of whether they were on psychotropic treatment^127–131^. The decrease in mean serum levels is apparent throughout the entire 24 h^128^. When comparing levels pre-effective and post-effective antipsychotic treatment, antipsychotics did not alter nocturnal melatonin secretion^129^. To note, three of four positive studies only included individuals with chronic schizophrenia. The one study that included both individuals with chronic schizophrenia and those who had just started experiencing psychotic symptoms found that the group whose symptoms had just started had increased nocturnal melatonin secretion compared to the individuals who were chronically ill^127^. The fourth study, by Afonso et al. (2011)^132^, which had negative findings, did not state whether the group of individuals with schizophrenia was suffering from chronic schizophrenia. Furthermore, Ferrier et al. (1982)^127^ pointed out that bodyweight also plays a role in melatonin secretion. In fact, when body weight was used as a covariate, the difference in melatonin levels between the cases and controls became insignificant^127^. Interestingly, when comparing the nocturnal plasma levels between individuals with schizophrenia and MDD, it was found that the levels in MDD are lower than those seen in schizophrenia^130^. There were no differences in the levels of melatonin in CSF^133^. Given melatonin’s role in sleep and the altered sleep patterns experienced by up to 78% of individuals with schizophrenia, melatonin research in the context of schizophrenia may be critical^125,134^.

#### Bipolar disorder (BD)

Early studies on plasma melatonin concentrations in BD patients suggested that there were no alterations^135^. However, preliminary evidence now suggests decreased serum melatonin levels among BD patients at all time points within a 24-h time period. When studied in different mood states, a significant decrease in melatonin levels of BD individuals in their depressed state was reported compared to healthy controls at 1 a.m. (peak melatonin onset) and in the early morning. Melatonin levels were only decreased in euthymic patients compared to healthy controls at 1 a.m. but no changes were found when comparing manic patients and healthy controls. No alterations in urinary melatonin levels were noted based on levels of 6SMT either^136^. More recent studies confirm decreased evening melatonin levels in saliva and CSF, but studies were unable to replicate the results in blood^124,137^. In fact, melatonin secretion in saliva was almost two times lower during habitual sleep onset in adolescents and young adults with BD compared to MDD^137^. The decreased melatonin levels may, in part, be explained by increased levels of interleukin-6, a pro-inflammatory cytokine, which induces monoamine oxidase A, which leads to an increase in the breakdown of serotonin, a precursor of melatonin^138,139^.

Although it cannot be said with certainty, one potential explanation for the decreases in melatonin among the three psychiatric disorders discussed could be genetic differences. More specifically, the genetics of melatonin synthesis. Two genes of importance are *AANAT* and *ASMT*, which encode enzymes responsible for converting serotonin into melatonin. Soria et al. (2010)^140^ identified two markers of *AANAT*, rs3760138 and rs4238969, both of which have allele and genotype (dominant model) frequency distribution differences between depressed patients (including unipolar and bipolar individuals) and healthy controls. Three haplotypes were also identified, two of which were protective against depression and one that was a susceptibility haplotype^140^. In other studies, markers of *ASMT* have also been linked to depression such as the ‘AA’ genotype of rs4446909 and the ‘GG’ genotype of rs5989681 being protective genotypes in two samples of individuals of Polish descent^141,142^. The study also reported differential mRNA expression levels in blood for *ASMT*, such that depression cases who had a ‘G’ allele for rs4446909 or a ‘G’ allele for rs5989681 had decreased mRNA expression levels^141^. In BD, there were allelic differences identified between cases and controls for markers of ASMT (‘G’ of rs4446909, ‘G’ of rs5989681, and ‘A’ of rs56690322) although only the finding for rs4446909 remained significant in an independent replication sample. A protective haplotype using the three markers already mentioned and rs6644635 was also identified. Individuals with the ‘GG’ genotype of rs4446909 showed lower enzymatic activity and mRNA levels^143^.

At this point, it is not clear what studies of tissue melatonin might reveal in these groups of patients and we can only speculate on their potential clinical significance. However, it would be expected that alterations of melatonin synthetic genes would affect both known pools of melatonin in a similar fashion.

## Conclusion

**Fig. 1:**
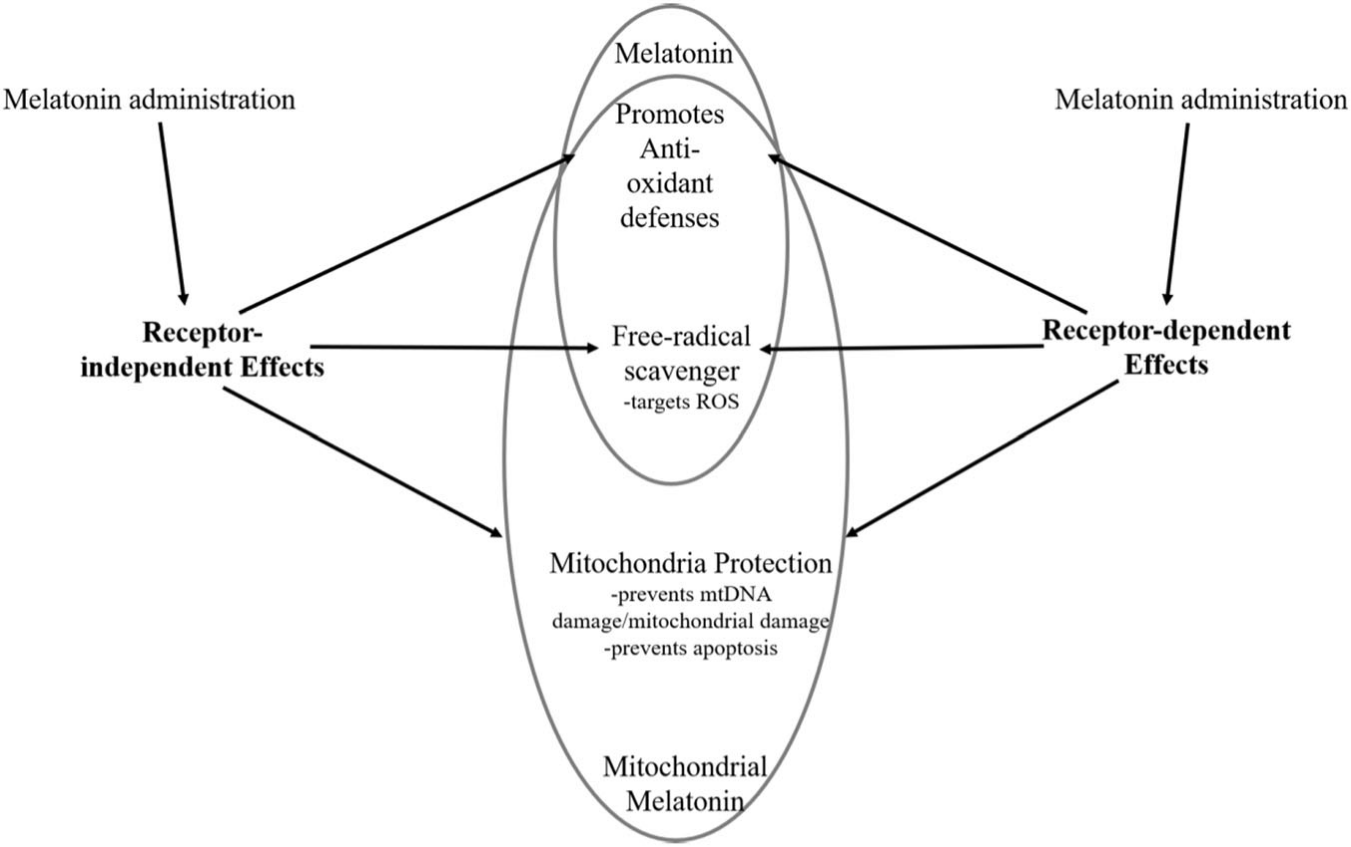
Overview of melatonin receptor-independent and receptor-dependent neuroprotective effects. Melatonin administration can elicit receptor-independent and dependent effects. Melatonin promotes anti-oxidant defenses and free-radical scavenging throughout the body. The relatively high levels of melatonin within mitochondria have the important benefit of enhanced protection against mtDNA damage and prevention of apoptosis.

There is no doubt that melatonin is an extremely versatile indoleamine, with the various roles and functions it has in the body. In addition to its well-known role as a hormone, a plethora of evidence has been put forth in support of its role as a neuroprotectant, immune modulator, and even as an antioxidant for the brain and body. We have provided a brief overview of some of these studies. For simplicity, we have created a diagram (Fig. 1) to summarize the neuroprotective properties of melatonin reviewed in this paper. The neuroprotective effects melatonin displays are similar between the receptor-independent and dependent pathways. Both pathways can promote antioxidant defenses, have free radical scavenging capabilities, and are able to protect mitochondria. Melatonin administration can also elicit its effects in a receptor-independent or dependent manner. Moreover, because of this newer role discovered for melatonin, it is important to investigate the implications it may have as a biomarker under different circumstances. Based on the vast amount of literature, decreased pineal and tissue melatonin appears to be a biomarker of aging. A reduction in pineal melatonin also appears to be a biomarker of psychiatric disorders, at least the three discussed in this review (MDD, schizophrenia, and BD) and may indicate the presence of neurodegenerative processes analogous to aging^144^.

Of concern for psychiatry, diagnostic criteria at present lump together patients in categories with varying pathological features. Subtyping these disorders should be done to take pathophysiological systems including melatonin into account in order to refine and tailor treatment. There are at least three causes of decreased melatonin: alterations in key melatonin synthetic genes as noted above; lessened availability of serotonin due to increased stress and proinflammatory cytokines that direct tryptophan down the kynurenine pathway and increases in light exposure during normal sleeping times^65^. These could reduce the neuroprotection seen in some patients. To avoid damaging degeneration, melatonin could be given as a treatment to restore neuroprotection.

As such, we recommend that future studies examine variations in genes involved in melatonin synthesis (for example ASMT), particularly in relation to the appearance of cognitive deficits in these psychiatric populations^140–143^. In addition, a measure of overnight 6SMT levels could also be relevant to estimate total body nocturnal melatonin via both its production and disposal. This can be accomplished by obtaining the first-morning sample of urine and determining the 6SMT level and normalizing it to the concentration of creatinine. Furthermore, treatment trials could readily be done on those with reduced melatonin with the aim of attempting to prevent deterioration of neuroprotection.

Finally, melatonin levels in mitochondria are about 100× higher than the levels found in the blood. When melatonin, and subsequently its protective actions are lacking, oxidative damage is remarkably high^79^. Therefore, the field should invest more effort in this powerful role of melatonin in controlling oxidative metabolism by examining, for example, the correlation between the levels of melatonin’s metabolites and markers for mitochondrial dysfunction or oxidative stress^145^.
